# Can Human Oral Mucosa Stem Cells Differentiate to Corneal Epithelia?

**DOI:** 10.3390/ijms22115976

**Published:** 2021-06-01

**Authors:** Sonia López, Lía Hoz, Eda Patricia Tenorio, Beatriz Buentello, Fátima Sofía Magaña, Ana Wintergerst, Alejandro Navas, Yonathan Garfias, Higinio Arzate

**Affiliations:** 1 Laboratorio de Biología Periodontal, Facultad de Odontología, Universidad Nacional Autónoma de México, CDMX 04510, Mexico; sonyletayf@comunidad.unam.mx (S.L.); liahoz@fo.odonto.unam.mx (L.H.); anawintergerst@comunidad.unam.mx (A.W.); 2 Departamento de Bioquímica, Facultad de Medicina, UNAM, Universidad Nacional Autónoma de México, CDMX 04510, Mexico; ep.tenorio@unam.mx; 3 Instituto de Oftalmología Conde de Valenciana, CDMX 06800, Mexico; bbuentello@institutodeoftalmologia.org (B.B.); fatima.magana@institutodeoftalmologia.org (F.S.M.); alejandro.navas@institutodeoftalmologia.org (A.N.)

**Keywords:** stem cells, cornea, epithelial differentiation, oral mucosa

## Abstract

Human oral mucosa stem cells (hOMSCs) arise from the neural crest, they can self-renew, proliferate, and differentiate to several cell lines and could represent a good source for application in tissue engineering. Because of their anatomical location, hOMSCs are easy to isolate, have multilineage differentiation capacity and express embryonic stem cells markers such as—Sox2, Oct3/4 and Nanog. We have used SHEM (supplemented hormonal epithelial medium) media and cultured hOMSCs over human amniotic membrane and determined the cell’s capacity to differentiate to an epithelial-like phenotype and to express corneal specific epithelial markers—CK3, CK12, CK19, Pan-cadherin and E-cadherin. Our results showed that hOMSCs possess the capacity to attach to the amniotic membrane and express CK3, CK19, Pan-Cadherin and E-Cadherin without induction with SHEM media and expressed CK12 or changed the expression pattern of E-Cadherin to a punctual-like feature when treated with SHEM media. The results observed in this study show that hOMSCs possess the potential to differentiate toward epithelial cells. In conclusion, our results revealed that hOMSCs readily express markers for corneal determination and could provide the ophthalmology field with a therapeutic alternative for tissue engineering to achieve corneal replacement when compared with other techniques. Nevertheless, further studies are needed to develop a predictable therapeutic alternative for cornea replacement.

## 1. Introduction

Stem cells have three main characteristics—they can self-renew, proliferate and differentiate to several cell phenotypes [1]. Mesenchymal stem cells (MSCs) are a fraction of adult cells that have the capacity to differentiate into adipocytes, osteoblasts, and chondrocytes, and they possess specific immune profiles. MSC canonical profiles must be positive to surface markers CD73, CD90 and CD105, and lack hematopoietic and endothelial markers like CD31, CD34 and CD45 [2,3]; these markers have been challenged in recent years because they are mostly specific to bone marrow-derived cells, but some of the markers identify most MSCs on any tissue in a perivascular niche, where they are usually found independently of their origin [3]. Mesenchymal tissues from the head and neck are special, in the sense that most of them derive from a mix of the neural crest (NC) and mesoderm, making it possible to obtain stem cells derived from NC that have the potential to form tissues from the region that they were originally isolated as well as tissues from other sites [4]. The oral cavity is easy to access and stem cells can be isolated from several sites such as—bone marrow, dental pulp of permanent and deciduous teeth, dental follicle, dental germ, apical papilla, oral mucosa, periosteum, periodontal ligament, salivary glands, and adipose tissues [5,6]. Of these sites, the oral mucosa is privileged since it can regenerate quickly and ages slowly in comparison to other tissues in the body. Both the epithelia and the lamina propria have different stem cells that represent important candidates for stem cell therapies [7]. A special population of stem cells from the lamina propria of the human oral mucosa (hOMSCs) is easily isolated and possesses great potential for differentiation. These cells express embryonic stem cell (ESC) markers; Tra2-49, Tra2-54, SSEA4, Oct4, Sox2 and Nanog, and the NC marker; nestin, besides the canonical MSC markers CD73, CD90, CD105 [8]. In vitro, they can differentiate into tissues of the three germ layers, and in vivo they form bilinear teratomas. These cells have been found to differentiate into NC lineages such as—astrocytes, dopaminergic neurons, cardiomyocytes, and osteoblasts [8,9,10,11,12,13].

Ophthalmology could benefit from tissue engineering. Corneal transplantation is the most frequently performed type of transplant worldwide, coming second only to kidney. In México it represented 54.2% of all transplant activity in 2019 [14].

The cornea forms part of the anterior ocular surface of the eye. It is a transparent and avascular tissue consisting of three cellular layers (epithelial, stroma and endothelial) and two acellular layers (Bowman membrane and Descemet membrane). The corneal epithelium comprises 10% of the total corneal thickness, absorbs nutrients and oxygen, while protecting the eye. The epithelium is squamous and non-keratinized with up to 5–6 layers, and composed of basal cells, wing cells as well as superficial cells [15]. The periphery with the conjunctiva is delimited by the limbus, which houses a unique population of cells called limbal epithelial stem cells (LESC) which renew the cornea epithelium [16,17]. The lack of these cells is called limbal epithelial stem cell deficiency (LESCD) and causes vascularization and opacification of the cornea. This deficiency elicits loss of eyesight. The etiology of LESCD can be—(1) congenital (aniridia, sclerocornea); (2) diseases with an extrinsic origin or trauma (chemical and thermal burns); (3) systemic diseases; and (4) idiopathic [18]. An autologous limbus transplant can help repair the cornea, but it has the risk of causing LESCD on the donating eye. Moreover, this option is applicable for people with only one eye damaged, and it does not have good results in people with an autoimmune disease. Another option is the allogenic transplant, which, beside complications like rejection, also faces lack of donors [19,20,21].

Using MSCs to replace cornea epithelia has been proposed [22,23]. The main limitations, however, are the cell source and their difficulty to transdifferentiate into epithelial cells. Because hOMSCs originate in the neural crest and have shown greater differentiation potential, we hypothesized that they have the ability to transdifferentiate into epithelia.

To facilitate the manipulation and transplant of the differentiated cells to the eye, we selected a scaffold that has been used in ophthalmology—the amniotic membrane. This membrane has been used to as an alternate therapy for superficial eye diseases when a cornea donor is not available or as a scaffold for LESC with success; tt facilitates epithelial cell migration because of its structure. Its stroma is similar to the cornea stroma, providing good biocompatibility and possesses anti-angiogenic, antibiotic, and anti-inflammatory properties [24,25].

## 2. Results

### 2.1. Isolation of the hOMSCs

**Figure 1:**
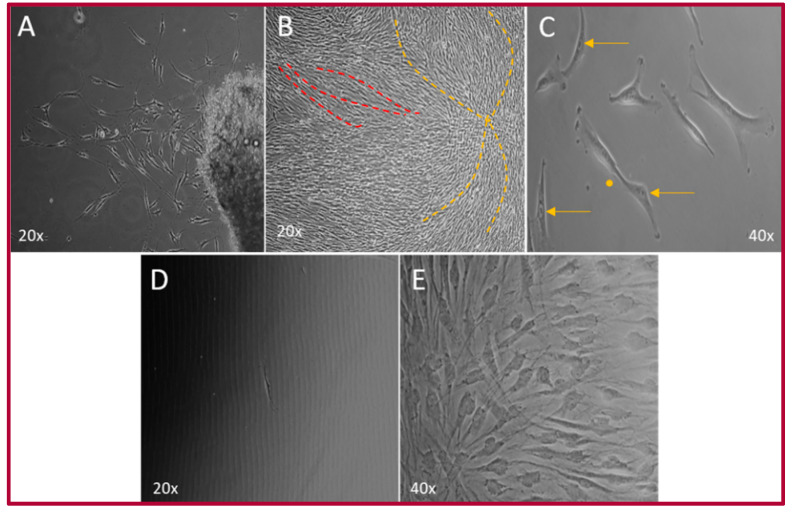
hOMSC culture. (**A**) Explant of oral mucosa, where cells can be observed migrating outward from the tissue fragment. (**B**) Cell culture at 90–100% confluence grouped in small currents (red lines) and in storiform pattern (yellow lines). (**C**) Individual cells, showing central nucleus with prominent nucleolus (yellow arrows). (**D**) Single cell culture at day 1. (**E**) Colony of cells showing a homogeneous morphologic phenotype.

The primary culture was established in approximately 31 days, after obtaining 1.4 cm^3^ tissue samples (Figure S1). The cells showed a fibroblast-like shape with central nuclei and a prominent nucleolus, with some cells in mitosis. When hOMSCs reached 90–100% confluence, cells started forming short currents and storiform (whirlpool) patterns. These cells also exhibited the capacity to form colonies from a single cell (Figure 1).

### 2.2. Cellular Characterization of hOMSCs

**Figure 2:**
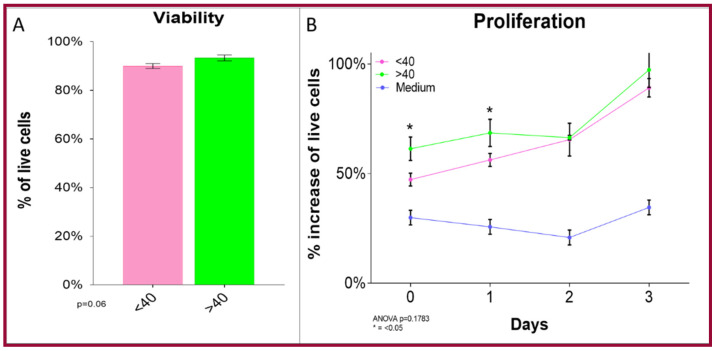
Comparison of hOMSCs among age groups. In samples obtained from healthy donors, (**A**) viability was over 90% for both age groups (<40 years old and >40 years old) with no significant difference between them. (**B**) In the proliferation assay, there was a slight difference on day one, but at day 3, there were no differences between age groups (<40 years old and >40 years old). (Bars represent mean ± SEM), *p* < 0.05 was considered significant, *n* = 3 per group).

Cellular number, viability and proliferation were determined by grouping the cells into two age groups from patients—either younger or older than 40. Our results revealed that hOMSCs from older patients show no differences with those from younger patients. No significant differences were observed in the number of cells or their viability among the groups (*p* = 0.06). There was a slight difference in the proliferation between both groups at day one of the culture, but there were no significant differences between groups at the end of the assay (*p* = 0.1763) (Figure 2).

**Figure 3:**
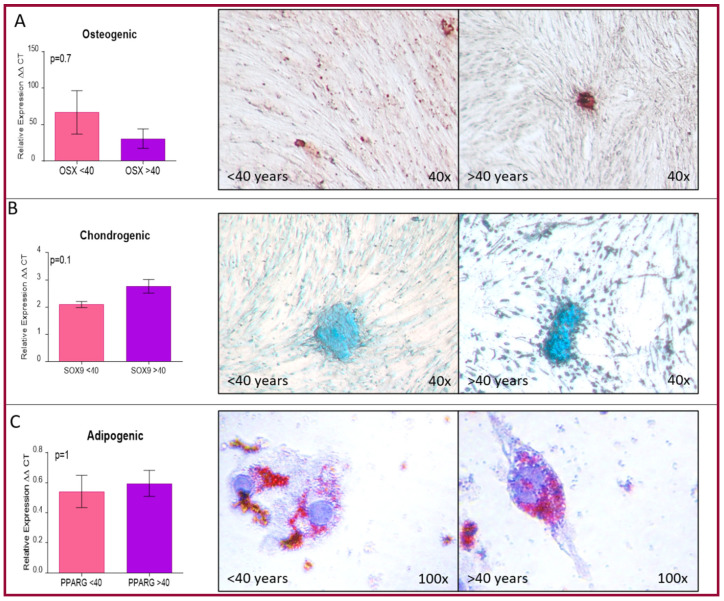
Comparison of hOMSC mesenchymal differentiation among age groups. (**A**) Osteogenic. Both age groups (<40 years old and >40 years old) showed an increase in OSX mRNA, a bone marker for osteoblasts as well as positive staining with alizarin red. (**B**) Chondrogenic. Both age groups (<40 years old and >40 years old) had an increased expression of Sox9 mRNA, a marker of chondrocytes and positive staining to alcian blue. (**C**) Although, there was no increase in PPARγ mRNA, a marker of adipocyte differentiation, the cells showed lipid vesicles stained with oil red O. (Bars represent mean ± SEM), *p* < 0.05 was the cut off for significance, *n* = 3 per group).

The same groups were used to confirm if there was any variation in their differentiation towards mesenchymal phenotypes—chondrogenic, osteogenic and adipogenic, in both cell populations. To confirm differentiation into these phenotypes, we used special stains specific for each one (alizarin red, alcian blue and oil red O) as well as RT-qPCR as markers that identify each one of them (Sox9, OSX, and PPARγ). The assays confirmed that both groups could differentiate into each phenotype, and more importantly that there were no significant differences between them (Figure 3). Since these two assays revealed that there are no differences between the groups, all further assays were performed randomly from passages one to four.

**Figure 4:**
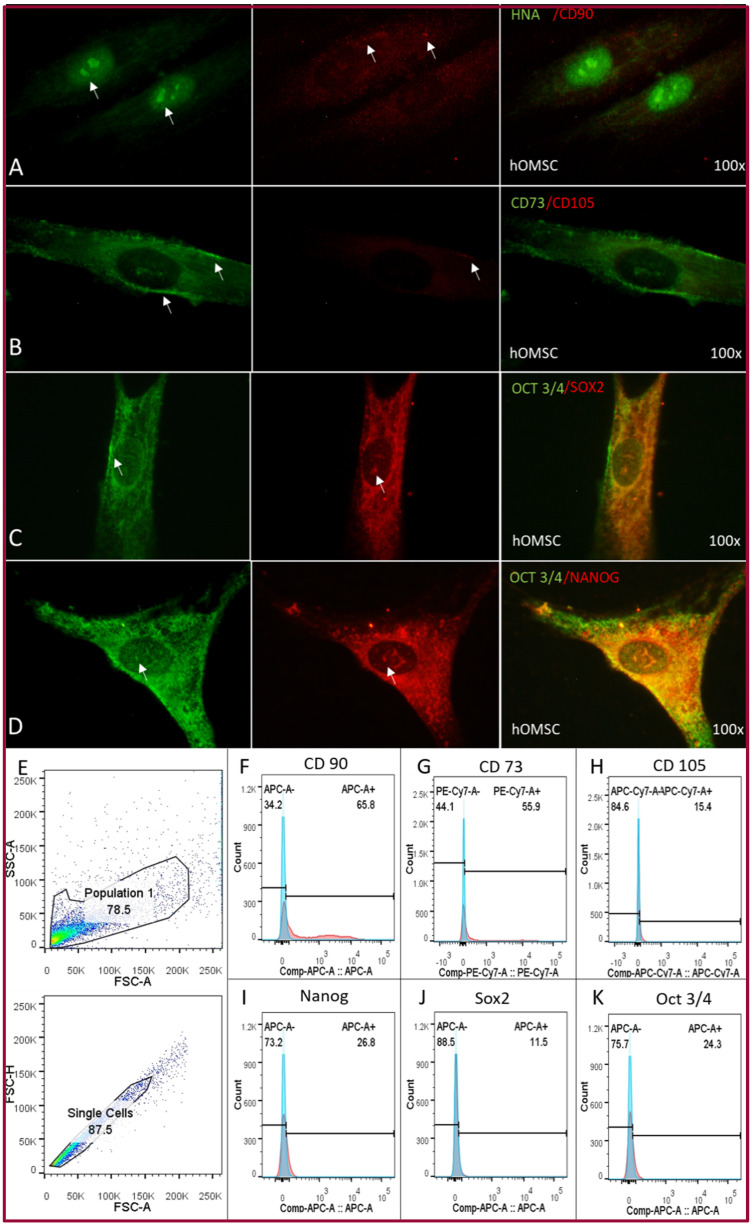
hOMSC characterization. (**A**–**D**) Immunofluorescence of hOMSCs and pluripotency markers. White arrows mark areas of positivity. (**E**) Flow cytometry gates. Percentage of positive cells in culture for hOMSCs—(**F**) CD90 65.8%, (**G)** CD73 55.9%, (**H**) CD105 15.4% and pluripotency markers—(**I**) Nanog 26.8%, (**J**) Sox2 11.5% and (**K**) Oct3/4, 24.3%.

The flow cytometry assay demonstrated the presence of mesenchymal stem cell markers—CD90, 65.8%; CD73, 55.9%; CD105, 11.5%; Nanog, 26.8%; Sox2 11.5% and Oct3/4 24.3% of the hOMSC culture. These markers were further confirmed by immunofluorescence. The localization of the markers was identified as follows—CD90 was present in the cytoplasm but the expression was low, CD73 was strongly expressed in the cell membrane, CD105 was weakly expressed in the cell membrane, Nanog, Oct3/4 and Sox2 were strongly expressed perinuclearly and inside the nucleus (Figure 4).

### 2.3. Epithelial Differentiation

**Figure 5:**
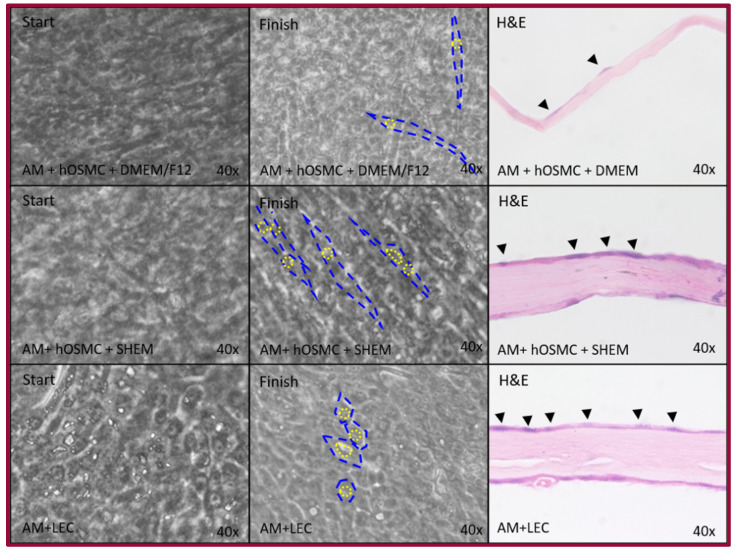
hOMSC epithelial differentiation. Comparison from the three conditions during the culture period and H&E staining yielded differences between groups. The culture with maintenance medium retained its fibroblast-like appearance with a prominent nucleus (black arrowhead) but almost no cytoplasm (AM + hOMSCs + DMEM/F12). The LEC line used as an epithelial control exhibited usual epithelial features—small, polygonal with a central nucleus and a 1:1 nucleus–cytoplasm ratio (AM + LEC). The experimental group displayed a fibroblast-like appearance with a prominent nucleus (black arrowhead); however, it was organized in palisades in the culture and showed an increase cytoplasm to nucleus ratio as observed in the H&E slides (AM + hOMSCs + SHEM). AM, amniotic membrane; hOMSCs, human oral mucosa stem cells; SHEM, supplemental hormonal epithelial medium; DMEM/F12, Dulbecco’s modified eagle medium/Ham’s F12.

Limbal epithelial cells (LECs) were used as a positive control. When LECs are expanded over amniotic membrane and they preserve their epithelial morphology—polygonal cells with a central nucleus and a cytoplasm with a nucleus ratio of 1:1. They were observed as rectangular cells with a central nucleus and abundant cytoplasm in the histological slides. hOMSCs were cultured over the amniotic membrane using the maintenance medium as a negative control. These cells have an elongated morphology, with a nucleus that can be in the middle of the cell or to one of the sides with a cytoplasm-nucleus ratio of approximately 3:1. The histological assessment revealed that the nucleus can be found over the amniotic membrane, and that there is almost no cytoplasm. In contrast, in the experimental group where hOMSCs were cultured over the amniotic membrane using the SHEM medium, which is the maintenance media for LEC, these cells preserved the elongated aspect of hOMSCs, but they were more organized and apparently had an increase in the cytoplasm–nucleus ratio. The cells were rectangular with an increased cytoplasm (Figure 5).

**Figure 6:**
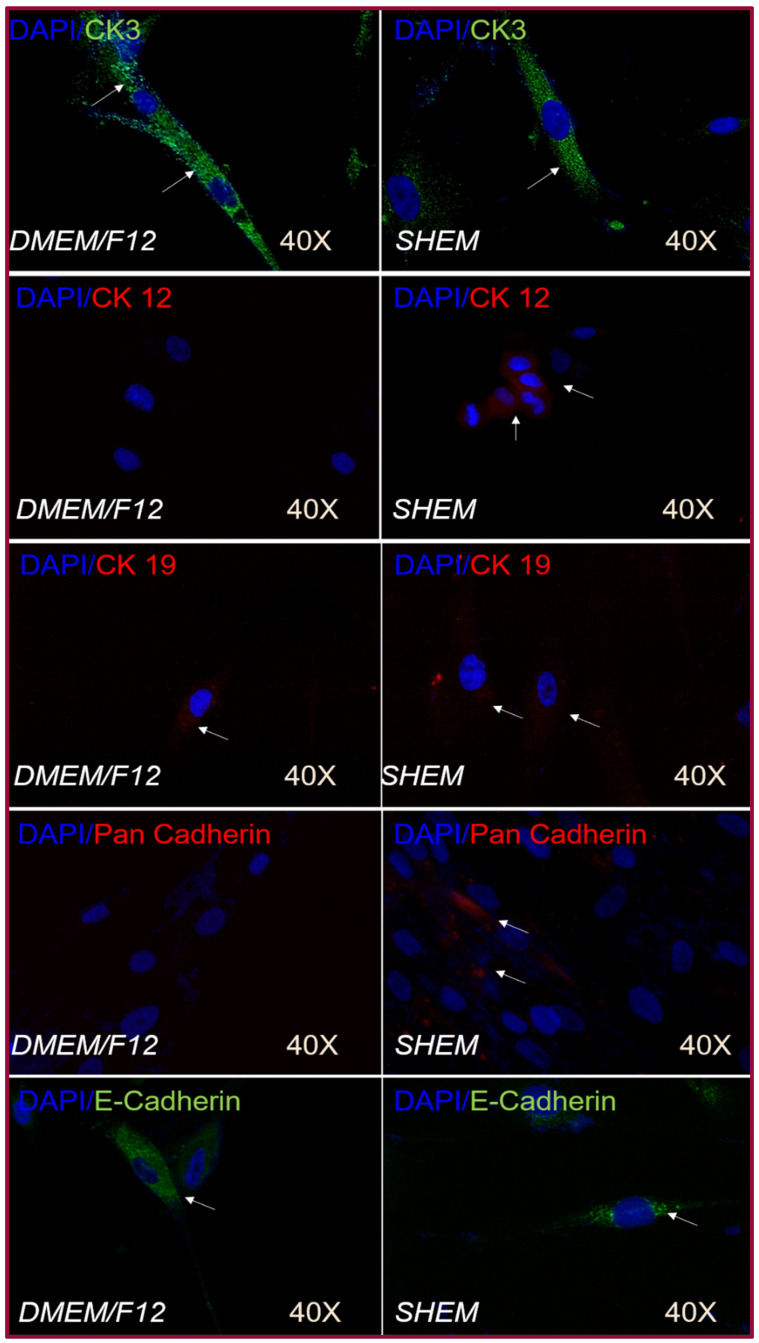
Epithelial markers—cell culture without AM. SHAM and DMEM/F12 groups presented intense positivity to CK3 in the cytoplasm (white arrows). The positivity to CK19 in both groups was low and localized in the cytoplasm (white arrows). E-cadherin was present in both groups, but the main difference was that DMEM/F12 medium showed diffuse cytoplasmic localization, but SHEM showed punctual localization in the cytoplasm (white arrows). hOMSCs cultured with SHEM expressed CK12 and Pan-cadherin (white arrows).

**Figure 7:**
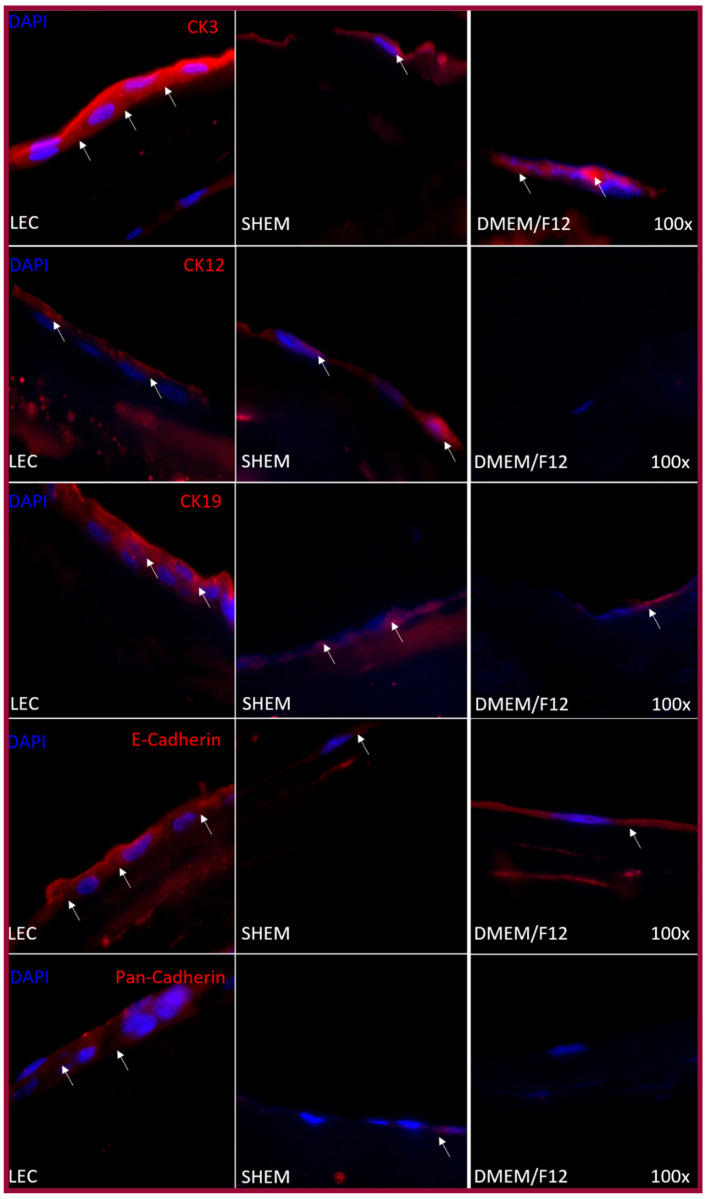
Epithelial marker expression with AM. Cytokeratin-3 was highly expressed in both groups when compared to the positive control (LEC). Cytokeratin-12 was completely absent in the DMEM/F12 group, and although the SHEM group expressed the marker, its expression was lower than with LEC. Cytokeratin-19 was expressed in all groups, but when compared to LEC, the expression in the DMEM/F12 group was lower than in the SHEM group. Pan-Cadherin was absent from the DMEM/F12 group and even though the SHEM group expressed the marker it was very modest in scattered cells. E-cadherin was expressed by all groups, but it was scattered in the cytoplasm of the cells in the DMEM/F12. The SHEM group positivity was mainly localized in the periphery of the nucleus.

Immunofluorescence was performed on hOMSCs cultured on coverslips and hOMSCs and LECs cultured on the amniotic membrane. hOMSCs cultured on coverslips using SHEM and maintenance medium were positive to CK3, CK19 and E-cad. They acquired CK12 and Pan-cad only when they were cultured with SHEM medium (Figure 6). The cells cultured on the amniotic membrane exhibited a similar expression pattern; LECs and hOMSCs cultured with SHEM and maintenance media were positive to CK3, CK19 and E-cad; acquired CK12 and Pan-cad were only expressed when cultured with SHEM media (Figure 7).

## 3. Discussion

Human oral mucosa stem cells are adult stem cells originating from the neural crest [26]. These cells express ESC markers such as Nanog, Sox2 and Oct3/4, which explain the plasticity observed in these cells. These markers are not just important markers of plasticity. However, the differences in expression the levels can also be used to track the capacity to differentiate into specific phenotypes [27,28,29,30]; therefore, in the case of eyes, cells positive to Sox2 and Pax6 (which act in a synergic manner to drive corneal differentiation in embryogenesis) might have a better chance of acquiring a corneal phenotype [31]. All this is important since they have advantages over cells isolated from other sites of the body and explains the capacity of these cells to differentiate into cell lineages outside their tissue of origin. This, as well as the fact that they are isolated from the lamina propria of the oral mucosa using local anesthesia and that age does not affect their viability, proliferation or differentiation, makes them good candidates to be used in tissue engineering since they do not require long periods for maintenance or prior storage [8].

Although there are cell-based therapies to treat LESCD with a certain success, it has limitations and risks. These therapies need a healthy eye to obtain the cells, therefore, patients with bilateral problems cannot be benefited. Even in patients that are candidates for this therapy, the risk of causing LESCD in the donor eye is high. Another limitation of this therapy relates to patients with autoimmune or dry eye diseases, since this therapy may not be successful in this type of patient, very likely because of the chronic inflammatory processes affecting the eye [23,32,33,34]. Mesenchymal stem cells have already been proposed to treat LESCD, and avoid harm to healthy eyes, thus giving patients with bilateral disease another option for treatment [22].

The results observed in this study show that hOMSCs possess the potential to differentiate toward epithelial cells. However, there is no consensus on how experiments to confirm cells’ capacity to transdifferentiate to epithelial cells should be analyzed. These cells express CK3, CK12 and CK19, which are markers associated with corneal epithelial cells [23]. The cornea is a complex tissue in which cell-to-cell contact plays a central role in keeping its structure. This contact is mainly mediated by E-cadherin, and even though hOMSC cells expressed this marker, the proper localization that will provide the graft with the needed resistance in vivo must be confirmed in subsequent studies [35,36,37]. Although obtaining an epithelial phenotype is a big step towards the generation of a corneal graft, it would not be complete without an LESC zone. These cells have self-renewal capacity and at the same time maintain the corneal structure by preserving the separation between the corneal space and the conjunctiva. Given the characteristics present in hOMSCs, a possible direction could be determining markers such as Pax6, which is the canonical marker for ocular development, as well as it’s regulator Wnt7a. Another important marker is ABCB5, which is present during the development and repair of the cornea, along with ΔNp63α, which is an isoform of p63, which is responsible for the LESC proliferation [23,38,39,40,41].

Trans-differentiation (the conversion of a differentiated cell type to another differentiated cell type) has always been a controversial subject. It is considered both a normal event in embryogenesis and repair, and a part of the reprograming of somatic cells into induced pluripotent stem cells [42]. Trans-differentiation can be achieved either by gene manipulation or handling the cell culture environment through enriched media. Recently, advances in the field of chemical trans-differentiation have been reported, giving to the field of tissue engineering multiple tools to work with [43,44]. This study shows the first approach towards application of hOMSCs for cornea tissue engineering. Most studies involving differentiation of MSCs towards cornea epithelium have used enriched media optimized for epithelium, however, the main concern of using genetic reprogramming is the appearance of a tumor in the short- or long-term after the therapy has been applied [23,43]. Epithelium mechano-transduction is also a trans-differentiation tool, where cadherins play an important role, since they can stiffen the cell, therefore provoking signals to transduce into the nucleus, influencing the epithelial phenotype. In our study, E-cadherin showed positivity. Nevertheless, is not localized in the cell membrane, and consequently, it neutralizes the cell to receive signals that will help express the epithelial phenotype. Nonetheless, future studies to determine the specific role of E-cadherin in the trans-differentiation of hOMSCs into epithelial cells should be performed [34,45,46,47].

Important for tissue engineering, especially for the cornea, is the scaffold used to generate a graft that will allow cells to maintain their differentiation potential as epithelial stem cells as well as to differentiate into the specific epithelia in cornea—a non-keratinized stratified squamous epithelium. This is a real challenge given that part of the differentiation potential of epithelial cells comes from the stiffness or softness of their environment. The stroma under the limbus is softer than the stroma under the differentiated cornea epithelium, which in part drives the replacement of the epithelium from the limbus to the central cornea [46,47]. The interaction with the stroma in epithelia is important because it can also drive differentiation towards other types of epithelia. Stiffer stroma caused by chronic inflammation may drive LESCs to express cytokeratins related to keratinized epithelium, which would be undesirable for corneal grafts, and has been observed when treating LESCD with LESC grafts, with grafts tending to fail in people with autoimmune diseases [24,34,47].

The amniotic membrane has been used with and without LESC culture because it presents anti-inflammatory, antiangiogenic, and antibiotic characteristics; it is also an ideal scaffold for LESCs. The amniotic membrane and cornea also share similarities in their structure. This membrane also provides cells with specific signals that promote epithelial differentiation [17,48,49]. In our study, the amniotic membrane allowed hOMSCs to adhere and when treated with SHEM medium, acquire an epithelial-like phenotype, as observed in hematoxylin and eosin-stained slides.

In conclusion, the use of MSCs for corneal replacement therapies yields controversial results [50]. Our study demonstrates that hOMSCs readily express markers of corneal determination and, when cultured under specific conditions, they express the molecular markers that are lacking, although more experiments and tests are needed to elucidate their true potential and limitations (this study’s greatest limitation is the use of microscopic observation only, and more analysis is needed like flow cytometry and western blot, to confirm the expression of epithelial-related molecules) and must be compared in efficiency and success with other techniques. Our results show that hOMSCs possess the therapeutic potential for cornea replacement.

## 4. Materials and Methods

### 4.1. Culture Media

#### Transport Media

DMEM (Dulbecco’s Modified Eagle Medium), antibiotics 3× (300 µg/mL streptomycin and 300 IU/mL penicillin, gentamicin 150-µg/mL, amphotericin B 3.75-µg/mL), sodium pyruvate 10 mM and 1:10 MEM non-essential amino acids 100×—Glycine, L-Alanine, L-Asparagine, L-Aspartic acid, L-Glutamic Acid, L-Proline, L-Serine 10 mM. Maintenance medium (DMEM)—DMEM, 5% to 20% of fetal bovine serum (FBS) of and antibiotics 1× (100 µg/mL streptomycin and 100 IU/mL penicillin, gentamicin 100 µg/mL, amphotericin B 2.5 µg/mL), sodium pyruvate 10 mM and 1:10 MEM non-essential amino acids 100×. Adipogenic medium—DMEM, SFB 10% antibiotics 1×, sodium pyruvate 10 mM, 1:10 MEM non-essential amino acids 100×, 0.5 mM isobutyl-methyl-xanthin, 0.5 mM indomethacin, 1 mM dexamethasone. Chondrogenic medium—DMEM, FBS 2%, antibiotics 1×, sodium pyruvate 10 mM, 1:10 MEM, 1 µg/mL of ascorbic acid, ITS-G 1 mL of stock at 100× (0.1721 mM Insulin, 0.0068 mM Transferrin, 0.0038 mM Sodium Selenite) (Sigma Aldrich, Toluca, Mexico), 10^−8M^ dexamethasone and 10 ng/mL hr-TGF (Sigma Aldritch, Toluca, Mexico). Osteogenic medium—DMEM, FBS 10% antibiotics 1×, 10 mM sodium pyruvate, 1:10 MEM non-essential amino acids 100×, 10 mM β-Glycerophosphate, 50 µg/mL ascorbic acid and 10^−8M^ dexamethasone. Maintenance medium 2 (DMEM/F12)—DMEM/F12 (Dulbecco’s Modified Eagle Medium 1:1 with Medium Ham 12), FBS at 5% or 10%, antibiotics 0.5× (50 µg/mL streptomycin and 50 IU/mL penicillin, gentamicin 50 µg/mL, amphotericin B 1.25 µg/mL), 10 mM sodium pyruvate and 1:10 MEM non-essential amino acids 100×. Epithelial Maintenance and Differentiation (SHEM)—DMEM/F12, FBS at 5%, antibiotics 0.5×, 0.5% dimethyl sulfoxide (DMSO), hEGF 2 ng/mL (Sigma Aldrich, Toluca, Mexico), ITS (Insulin 5 µg/mL, Transferrin 5 µg/mL, Sodium Selenite 5 ng/mL) (Sigma Aldritch, Toluca, Mexico), hydrocortisone 0.5 µg/mL, cholera toxin A (TCA) (Sigma Aldritch, Toluca, Mexico) 30 ng/mL. Media reagents unless otherwise stated were obtained from Thermo Fisher Scientific, Mexico City, Mexico).

### 4.2. hOMSCs Isolation

Oral mucosa was obtained from 14 healthy patients (19–68 years) that required a third molar surgery. The project was approved by the Ethics Committee of the School of Dentistry of the Universidad Nacional Autónoma de México (CIE/0303/02/2018) and inform consent was obtained from the participants. Samples were washed thoroughly with DMEM media supplemented with antibiotics as described above, macerated, and placed in a solution of dispase II 2 mg/mL and type III collagenase 2 mg/mL (Thermo Fisher Scientific, Mexico City, Mexico) for 2 h at 37 °C. They were vortexed every 15 min and placed on 25 cm^2^ culture flasks. Media were changed every other day until reaching 90% confluence and then replated on 75 cm^2^ culture flasks. Single cell cultures were also performed to test the clonogenic capacity of the cells. Passages 2 through 7 were used for the experiments. To increase the cell number prior to the experiments, the cells were plated in 75 cm^2^ culture flasks in the corresponding maintenance media. Cultures were kept at 37 °C, with an atmosphere of 5%CO_2_, 95%O_2_ and 100% humidity.

### 4.3. Limbal Epithelial Cells

Corneoscleral rims were obtained from cadaveric donor corneas. They were washed with phosphate buffered saline pH 7.4 (PBS). The iris, conjunctiva and endothelium were removed. The rims were placed in dispase II 2 mg/mL (Thermo Fisher Scientific, Mexico City, Mexico) for 40 min at 37 °C, washed with PBS and cut in fragments of approximately 2 mm. These fragments were cultured directly over the amniotic membrane with the epithelia side up; one drop of fetal bovine serum (FBS) was placed over the fragment for 8 h and then the epithelial medium was added.

### 4.4. Cellular Characterization

#### 4.4.1. Cell Number and Viability

hOMSCs at passage 2 from every subject were cultured up to 90% confluency in 75 cm^2^ tissue culture flasks. They were trypsinized with trypsin 0.25%-EDTA (Thermo Fisher Scientific, Mexico City, Mexico) for 2 min at 37 °C, de-attached and then centrifuged, washed, and placed on 1 mL of serum-free media. Cells were counted using 10 µl of cell solution and 10 µl of tripane blue in a countess device (Thermo Fisher Scientific, Wilmington, DE, USA) according to the manufacturer’s recommendations. The number of live and dead cells as well as viability were determined. Results are expressed as the mean ± SD (<40 years vs. >40 years).

#### 4.4.2. Cell Proliferation

Cell Proliferation was determined using the Presto Blue Kit (Thermo Fisher Scientific, Wilmington, DE, USA) following the manufacturer’s instructions. On a 96-well plate, 5 × 10^3^ cells/well were plated, kept for 24 h to allow attachment. Maintenance medium was collected, and the cells were placed on a mix of 90 µl with 10 µl Presto Blue reactive and incubated at 37 °C 5% CO_2_, 95%O_2_ and 100% humidity. After 2 h, two measures (A_570_ and A_600_) were taken at 24, 48, 72 and 96 h according to the manufacturer’s instructions. Wells without cells were used as a negative control; the value of these wells was subtracted from the wells with cells. Results are expressed as the mean ± SD (<40 years vs. >40 years).

#### 4.4.3. Mesenchymal Differentiation

Six/well plates were used for each phenotype; 1 × 10^4^ cells/well were plated and left overnight to attach, then cultured with osteogenic, chondrogenic or adipogenic differentiation media for 21 days. Two wells of each plate were fixed with PFA and stained with 2% alizarin red, alcian blue, and oil red O.

#### 4.4.4. RT-qPCR

Total RNAs were extracted according to the manufacturer’s recommended protocols with Trizol Reagent (Invitrogen, Carlsbad, CA, USA). RNA was quantified with a Nanodrop Spectrophotometer (Thermo Fisher Scientific, Wilmington, DE, USA). Ten nanograms were used per reaction and the level of mRNA expression was quantified by the one-step real-time RT-PCR method using a SuperScript^®^ III Platinum^®^ SYBR^®^ Green One-Step qPCR Kit (Invitrogen, Carlsbad, CA, USA). A 25-μL reaction was set up with the following PCR conditions—(cDNA synthesis) 50 °C for 3 min, denaturation at 95 °C for 5 min followed by 40 cycles of 95 °C for 15 s, 60 °C for 30 s, and finally 40 °C for 1 min. Amplifications were performed in a Corbett Rotor-Gene 6000 (Qiagen, Valencia, CA, USA). All experiments were performed in triplicate and expression levels were obtained using minus delta-delta Ct method normalizing for GAPDH. Primer sequences are reported in Table 1.

#### 4.4.5. Immunofluorescence

Cells (1 × 10^3^) were plated and left to adhere in 12 mm coverslips previously coated with Poly Lysine-L for 24 h; then washed with PBS and fixed with 1% paraformaldehyde (PFA) for 20 min and then washed twice with PBS. They were incubated in a humid chamber with a 1:50 dilution of the primary antibodies—CD105 (goat anti-human), CD90 (goat anti-human); Sox2 (rabbit anti-human), Nanog (rabbit anti-human); Oct3/4 (mouse anti-human), CD73 (mouse anti-human) (Santa Cruz Biotechnology, Dallas, TX, USA), HNA (mouse anti-human) (Abcam PLC, Cambridge, UK) in BSA 1%. Coverslips were incubated at 4 °C overnight, washed twice with PBST (PBS + 0.1% Tween20) and twice with PBS. Secondary antibodies were diluted 1:100 (goat anti-mouse FITC), 1:400 (goat anti-rabbit-Alexa 546), and 1:400 (rabbit anti-goat-Texas Red) (Santa Cruz Biotechnology, Dallas, TX, USA), and then incubated at room temperature for 2 h, washed 1× with PBST and 2× with PBS. They were mounted on slides using glycerol diluted in PBS (1:2). Negative controls were performed using secondary antibodies in the absence of the primary antibody (Figure S2).

#### 4.4.6. Flow Cytometry

hOMSCs were trypsinized with Trypsin–EDTA 0.25%, washed and counted. We used 2 × 10^5^ for each marker. Two protocols were used—for the superficial markers CD73-PeVio770 (clone REA804), CD90-APC (clone REA897), CD105-APCVio770 (clone REA794) (Miltenyi Biotec, Bergisch Gladbach, Germany) hOMSCs were washed in cold buffer (PBS + BSA 0.5% + EDTA 2 mM), antibodies were diluted 1:50 in the same buffer and incubated for 30 min at 4 °C in a dark environment, they were then washed in cold buffer and fixed using 1% PFA for 10 min, washed and resuspended in cold PBS. For the intracellular markers Nanog-APC (clone REA314), Oct3/4-APC (clone REA622) and Sox2-APC (clone REA320) (Miltenyi Biotec, Bergisch Gladbach, Germany), a stain kit FOXP3 (Miltenyi Biotec, Bergisch Gladbach, Germany) was used as recommended by the manufacturer. The cells were suspended in the permeabilization/fixation buffer for 30 min at 4 °C in darkness, washed with cold buffer and washed again twice with permeabilization buffer. The cells were resuspended again in 100 µl of permeabilization buffer with 10 µl of antibody and incubated for 30 min at 4 °C in a dark environment, then washed twice in permeabilization buffer and once with cold buffer and at last in PBS. They were read immediately using a BD flow cytometer (BD, Franklin Lakes, NJ, USA).

### 4.5. Epithelial Differentiation

#### 4.5.1. Epithelial Differentiation

Three amniotic membranes were obtained from the Amniotic Tissue Bank of the Insitute of Ophthalmology Conde de Valenciana, after elective cesarean section with informed consent from the donor, and were preserved at –80 °C until used as previously reported [51], washed twice in PBS, and placed on 2 mg/mL of dispase II (Thermo Fisher Scientific, Mexico City, Mexico) for 40 min with the epithelial side towards the Petri dish. At the end they were washed with PBS. The epithelium was mechanically removed by softly rubbing the membranes with a sterile cotton swab. The PC membrane was removed from the trans-well wells and substituted by the amniotic membrane attached with a rubber band. Three conditions were tested—(1) hOMSCs with DMEM/F12; (2) hOMSCs with SHEM; (3) LEC and hOMSCs were plated simultaneously on coverslips with conditions 1 and 2. They were kept until confluence, changing the media every other day. Membranes and coverslips were then processed for histological analysis and immunofluorescence.

#### 4.5.2. Immunofluorescence was Performed as Previously Described

hOMSCs were incubated in a humid chamber with a 1:100 dilution of primary antibodies—CK3 (rabbit anti-human) CK12 (goat anti-human), CK19 (mouse anti-human) (Santa Cruz Biotechnology, Dallas, TX, USA), Pan-Cadherin (mouse anti-human) E-cadherin (mouse anti-human) (Abcam PLC, Cambridge, UK). Secondary antibodies were diluted 1:800—goat anti-rabbit-FITC and goat anti-mouse-Alexa 594 (Santa Cruz Biotechnology, Dallas, TX, USA). Slow Fade Gold Antifade Mountant with DAPI (Thermo Fisher Scientific, Wilmington, DE, USA) was used to mount all coverslips. Negative controls were performed by incubating secondary antibodies in the absence of the primary antibody (Figure S2).

#### 4.5.3. Histology and Immunofluorescence

Membranes were fixed in Bouin’s solution, embedded in paraffin, sectioned at 5 µm thickness, and mounted on glass silanized slides. The sections were deparaffinized in descending concentrations of ethanol and distilled water and stained with hematoxylin and eosin (H&E). For the immunofluorescence, marker slides were blocked with BSA 1% (albumin 1% in PBS) for 1 hr, washed twice in PBST (Tween 20 0.1% in PBS) and incubated in a humid chamber at a 1:100 dilution of the primary CK3 (rabbit anti-human) CK12 (goat anti-human), CK19 (mouse anti-human) (Santa Cruz Biotechnology, Dallas, TX, USA), Pan-Cadherin (mouse anti-human) E-cadherin (mouse anti-human) (Abcam PLC, Cambridge, UK) for 2 h, washed twice in PBST and once in PBS. Secondary antibodies were diluted 1:800; goat anti-mouse-Alexa 594 and goat anti-rabbit-Alexa 546 (Santa Cruz Biotechnology, Dallas, TX, USA). Slow Fade Gold Antifade Mountant with DAPI (Thermo Fisher Scientific, Wilmington, DE, USA) was used to mount all coverslips. Negative controls were performed by incubating secondary antibodies in the absence of the primary antibody (Figure S2).

### 4.6. Microscopic Analysis

Photomicrographs were taken using an Apotome II microscope (Carl Zeiss, Mexico City, Mexico) and proccessed using the software Zen [52], parameters were set using a negative control, for each filter combination, and all photomicrographs were analyzed after processing with the set parameters, to standarized autoflouorescence and background noise. For immunofluorescence stains, the photomicrographs were analyzed by two independent observers. For H&E stains, the slides were analyzed and described by an oral pathologist.

### 4.7. Statistical Analysis

Results are expressed as mean ± SE. For viability and RT-qPCR, T tests were used to analyze differences between two groups, two-way ANOVA was used to analyze overall differences in cell proliferation and Bonferrioni’s post hoc test to search for differences between pairs of data sets. All analyses were performed using GraphPad Prism 8 [53].

## Figures and Tables

**Table 1:** Primer Sequences for RT-qPCR.

Gene	Sequence 5′-3′		Size (nt)	Tm (°C)
*SOX9*	F	GTA ATC CGG GTG GTC CTT CT	20	58.8
	R	GAC GCT GGG CAA GCT CT	17	59.68
*OSX*	F	GCC AGA AGC TGT GAA ACC TC	20	59.12
	R	GCT GCA AGC TCT CCA TAA CC	20	58.98
*PPAR-γ*	F	GAG AGA TCC ACG GAG CTG AT	20	58.67
	R	AGG CCA TTT TGT CAA ACG AG	20	56.91
*GAPDH*	F	CAACGGATTTGGTCGTATTGG	21	59.4
	R	GCAACAATATCCACTTTACCAAGAGTTAA	29	59.5

## Data Availability

The data presented in this study are available on request from the corresponding author.

## Supplementary Materials

The following are available online at https://www.mdpi.com/article/10.3390/ijms22115976/s1.

## Abbreviations

MSC	Mesenchymal stem cells
NC	Neural crest
hOMSCs	Human oral mucosa stem cells
LESC	Limbal epithelial stem cells
LESCD	Limbal epithelial stem cells deficiency
RT-qPCR	Reverse transcriptase–real time polymerase chain reaction
LEC	Limbal epithelial cells
SHEM	Supplemental hormonal epithelium medium
DMEM/F12	Dulbecco’s Modified Eagle medium/Ham 12 medium
ESC	Embryonic stem cells
MEM	Minimum essential medium
FBS	Fetal bovine serum
hEGF	Human epidermal growth factor
DMSO	Dymethylsulfoxide
ITS	Insulin- transferrin-selenium
PBS	Phosphate buffered saline
EDTA	Ethylenediaminetetraacetic acid
PFA	Paraformaldehyde
PBST	PBS + 0.1% Tween 20
BSA	Bovine serum albumin
PC	Polycarbonate
