# Supplementary material for: Can Human Oral Mucosa Stem Cells Differentiate to Corneal Epithelia?
Source: Int J Mol Sci. 2021 Jun 1;22(11):5976. doi: 10.3390/ijms22115976 (PMC8198937; doi:10.3390/ijms22115976)
Supplement: Supplementary file 1 [file ijms-22-05976-s001.zip › ijms-1190323-supplementary.pdf]

SUPPLEMENTARY MATERIAL

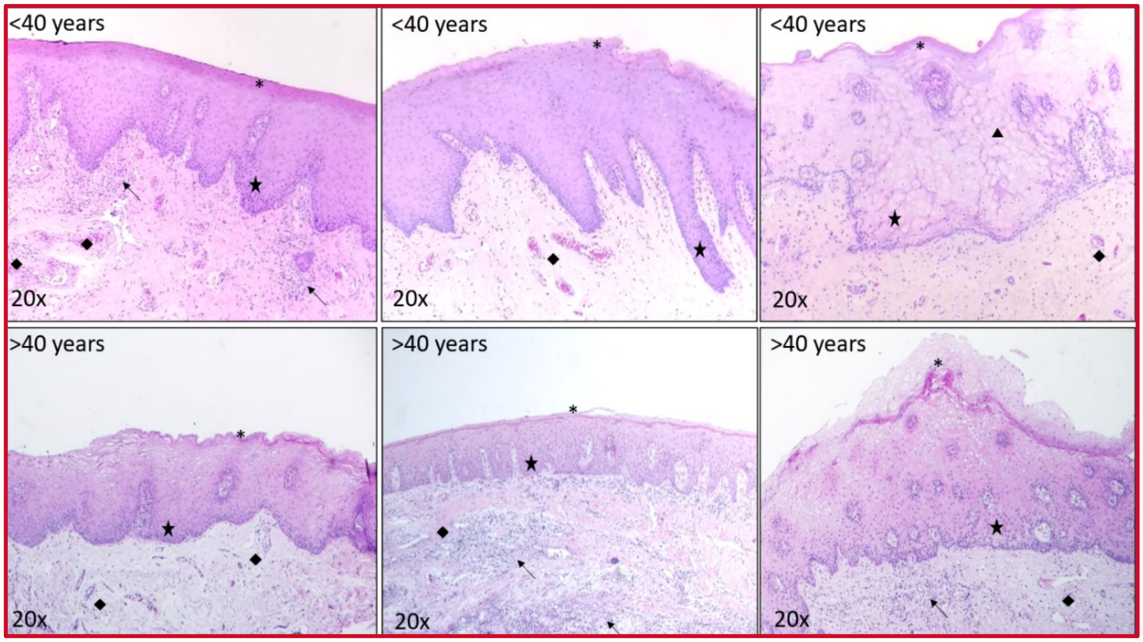

Supplementary Figure 1. Normal mucosa. Biopsies from patients taken for hOMSC isolation, showing stratified parakeratinized epithelium (star), with parakeratin (asterisk), some showed intracellular edema (triangle), normal vascularization (rhombus), and mild to moderate chronic inflammatory infiltrate showing a diffuse or patch pattern (arrow).

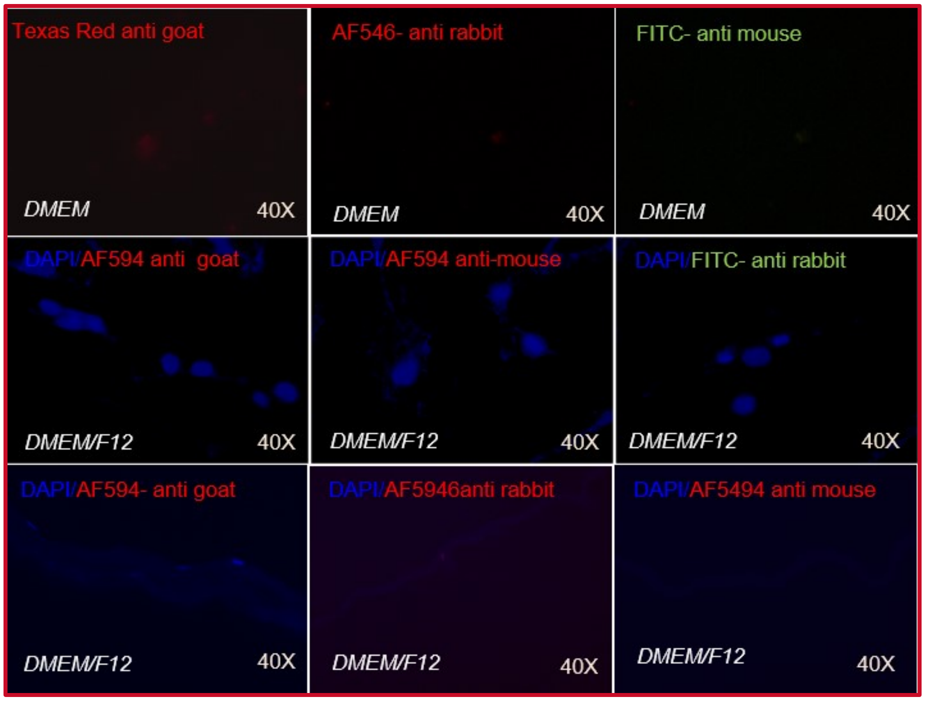

Supplementary Figure 2. Negative controls for immunofluorescence
